# Breast-feeding and maternal risk of type 2 diabetes: a prospective study and meta-analysis

**DOI:** 10.1007/s00125-014-3247-3

**Published:** 2014-05-01

**Authors:** Susanne Jäger, Simone Jacobs, Janine Kröger, Andreas Fritsche, Anja Schienkiewitz, Diana Rubin, Heiner Boeing, Matthias B. Schulze

**Affiliations:** 1 Department of Molecular Epidemiology, German Institute of Human Nutrition Potsdam-Rehbruecke, Arthur-Scheunert-Allee 114-116, 14558 Nuthetal, Germany; 2 German Center for Diabetes Research (DZD), Germany, http://www.dzd-ev.de/en; 3 Department of Internal Medicine, Division of Endocrinology, Diabetology, Nephrology, Vascular Disease and Clinical Chemistry, University Hospital Tübingen, Tübingen, Germany; 4 Department of Epidemiology and Health Monitoring, Robert Koch Institute, Berlin, Germany; 5 Lipid Clinic at the Interdisciplinary Metabolism Center, Charité University Medicine Berlin, Virchow Clinic Campus, Berlin, Germany; 6 Department of Epidemiology, German Institute of Human Nutrition Potsdam-Rehbruecke, Nuthetal, Germany

**Keywords:** Adiponectin, Breast-feeding, Epidemiology, HDL-cholesterol, Meta-analysis, Triacylglycerols, Type 2 diabetes, Weight, Women’s health

## Abstract

**Aims/hypothesis:**

We aimed to examine the association between breast-feeding and maternal risk of type 2 diabetes and to investigate whether this association is mediated by anthropometric and biochemical factors.

**Methods:**

A case–cohort study nested within the European Prospective Investigation into Cancer and Nutrition (EPIC)-Potsdam Study between 1994 and 2005 including 1,262 childbearing women (1,059 in a random sub-cohort and 203 incident cases) mainly aged between 35 and 64 years at baseline was applied. Self-reported lifetime duration of breast-feeding was assessed by questionnaire. Blood samples were used for biomarker measurement (HDL-cholesterol, triacylglycerols, C-reactive protein, fetuin-A, γ-glutamyltransferase, adiponectin). A systematic literature search and meta-analysis was conducted of prospective cohort studies investigating breast-feeding and risk of type 2 diabetes.

**Results:**

The HR for each additional 6 months of breast-feeding was 0.73 (95% CI 0.56, 0.94) in EPIC-Potsdam. Meta-analysis of three previous prospective studies and the current study revealed an inverse association between breast-feeding duration and risk of diabetes (pooled HR for lifetime breast-feeding duration of 6–11 months compared with no breast-feeding 0.89; 95% CI 0.82, 0.97). Adjustment for BMI and waist circumference attenuated the association (HR per six additional months in EPIC-Potsdam 0.80; 95% CI 0.61, 1.04). Further controlling for potentially mediating biomarkers largely explained this association (HR 0.89; 95% CI 0.68, 1.16).

**Conclusions/interpretation:**

Longer duration of breast-feeding may be related to a lower risk of diabetes. This potentially protective effect seems to be reflected by a more favourable metabolic profile; however, the role of body weight as a mediator or confounder remains uncertain.

**Electronic supplementary material:**

The online version of this article (doi:10.1007/s00125-014-3247-3) contains peer-reviewed but unedited supplementary material, which is available to authorised users.

## Introduction

Positive effects of breast-feeding have mostly been attributed to the health of the child [1]. Other findings suggest that mothers can benefit from breast-feeding as well [2, 3]. Although three prospective studies observed an inverse association between prolonged breast-feeding and the incidence of type 2 diabetes [4, 5], data are still scarce, and the underlying mechanisms by which breast-feeding lowers diabetes risk remain unclear. During gestation, enormous changes occur in women’s metabolism to ensure sufficient supply to the fetus. Breast-feeding is thought to ‘reset’ these metabolic changes [6]. Exclusive breast-feeding is accompanied by an increased energy requirement of about 2,000 kJ/day [7], but the impact of breast-feeding on longer-term weight development post partum remains unclear [8–10]. Studies also suggest higher insulin sensitivity and glucose tolerance in breast-feeding women [7, 9]. Although these observations contribute to the hypothesis that breast-feeding reduces the risk of type 2 diabetes, systematic evaluation of different pathways is so far lacking.

Our aims were therefore threefold: first, we evaluated the association between breast-feeding and diabetes risk in a large prospective study; second, we systematically reviewed other prospective studies evaluating this hypothesis; third, we investigated possible mechanisms underlying the association between breast-feeding and maternal risk of type 2 diabetes by evaluating a large set of diabetes-related markers reflecting insulin sensitivity or lipid metabolism as well as markers of liver fat accumulation and inflammation.

## Methods

### Study population

**Fig. 1:**
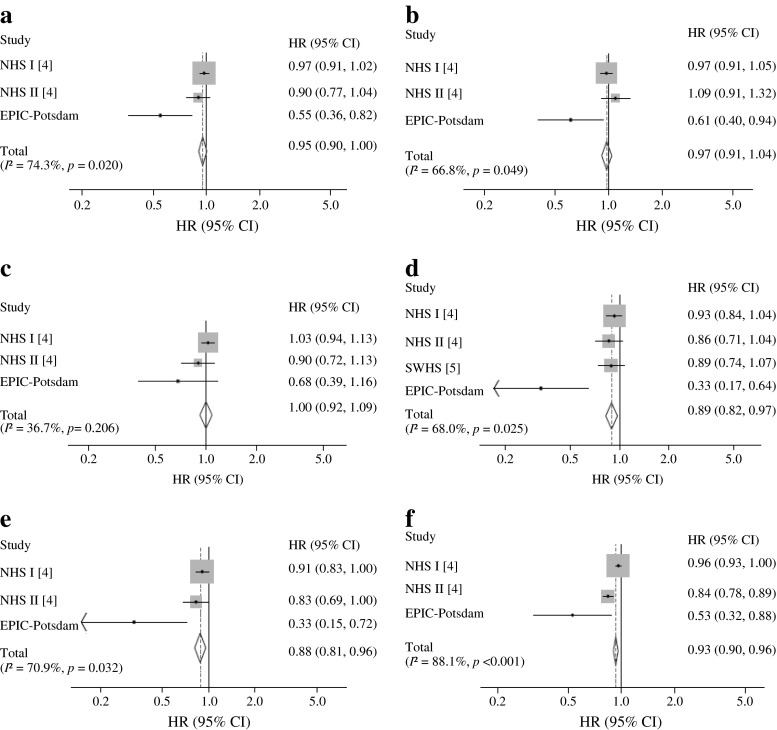
HRs (95% CIs) for association between duration of breast-feeding and type 2 diabetes. Categories of breast-feeding duration (compared with no breast-feeding): breast-feeding vs non-breast-feeding (**a**), >0 to 3 months (**b**), >3 to 6 months (**c**), >6 to 11 months (**d**), >11 to 23 months (**e**), per additional year (**f**). Models are adjusted for potential confounders. NHS, Nurses’ Health Study; SWHS, Shanghai Women’s Health Study

The European Prospective Investigation into Cancer and Nutrition (EPIC)-Potsdam Study has 27,548 participants (16,644 women and 10,904 men). The recruitment took place between 1994 and 1998 and focused on the general population in Potsdam and the surrounding area. All participants provided informed consent, and permission was given by the ethics committee of the State of Brandenburg, Germany. The baseline examination involved a personal interview including questions on prevalent diseases and women’s health, a self-administered questionnaire about socioeconomic and lifestyle characteristics and number of births including breast-feeding, interviewer-conducted anthropometric measurements and blood sample collection [11]. We used a case–cohort design (electronic supplementary material [ESM] Fig. 1) to evaluate biochemical risk factors for diabetes, described in detail previously [12]. From 26,444 participants who provided blood samples at baseline, 2,500 individuals were randomly selected. Of 1,430 women within this random sub-cohort, 1,301 reported having given birth. We excluded women with missing data on breast-feeding behaviour (*n* = 17), missing data on oral contraceptive use, implausible energy intake (<3,349 or >25,121 kJ/day) and prevalent diabetes including gestational diabetes mellitus (GDM), as well as women with missing data on BMI at the age of 25 years and missing biomarker measurements, leaving 1,059 women for analysis in the sub-cohort. After application of similar exclusion criteria, 226 incident cases identified among all childbearing mothers in the cohort remained for analysis (overlap of 23 women with the sub-cohort due to the case–cohort design).

### Assessment of breast-feeding duration and covariates

ESM Fig. 2 illustrates the time points of data collection and exposures.

Women recalled their age at childbirth and whether and for how long they breast-fed their children, separately for their first, second, third and last child (women with more than three children) in a self-administered questionnaire at the baseline examination. Eleven categories were given: 1 week or less; 2–3 weeks; 4–5 weeks; 6–7 weeks; 2 months; 3 months; 4–5 months; 6–7 months; 8–9 months; 10–11 months; 12 months or more.

Age at baseline examination and socioeconomic and lifestyle factors such as marital status, level of education, occupation, smoking behaviour and physical activity were assessed by a self-administered questionnaire and a personal interview. Weight, height and waist circumference at baseline examination were measured by trained interviewers who followed standard protocols under strict quality control. BMI at the age of 25 years was calculated from body weight at the age of 25 years (self-reported at baseline) and measured height. Dietary intake during the preceding 12 months was assessed through a validated food frequency questionnaire.

### Determination of biomarkers

We used a large set of biomarkers reflecting insulin sensitivity and lipid metabolism as well as markers of liver fat accumulation, such as γ-glutamyltransferase and fetuin-A, and C-reactive protein (CRP) as an inflammation-related marker.

Biomarkers were measured in blood samples collected at the baseline examination and stored at −80°C or lower until analysis. Plasma CRP concentrations were measured with a high-sensitivity latex-enhanced immunoturbidimetric assay on an automatic Advia 1650 Analyzer (Siemens Medical Solutions, Erlangen, Germany). Plasma adiponectin concentrations were determined by ELISA (Linco Research, St Charles, MO, USA). Plasma levels of triacylglycerols, HDL-cholesterol, γ-glutamyltransferase and fetuin-A were measured with the automatic Advia 1650 Analyzer. For determination of fetuin-A, an immunoturbidimetric method was used with specific polyclonal goat antibodies to human fetuin-A (BioVendor Laboratory Medicine, Modreci, Czech Republic) [12]. All assay procedures were performed according to the manufacturer’s description. LDL-cholesterol levels were calculated using the Friedewald equation [13].

### Assessment of type 2 diabetes

Every 2–3 years, follow-up questionnaires were sent out to identify incident cases of diabetes. All incident cases were verified by treating physicians, who were asked in a questionnaire to provide data on the date and type of diagnosis, diagnostic tests and the treatment. Cases confirmed by a physician (ICD-10: E11) and a diagnosis date after the baseline examination were considered to be confirmed incident cases of type 2 diabetes. For the present analysis, we used data collected until August 2005. Women with missing follow-up questionnaires were excluded from the analysis. However, the follow-up rate was high, exceeding 95%, and was similar between breast-feeding categories (data not shown).

### Statistical analysis

Lifetime breast-feeding duration was calculated as the sum of breast-feeding periods for each child and was stratified into five categories: no breast-feeding; ≤3 weeks; >3 weeks to <2 months; ≥2 months to <6 months; ≥6 months.

To evaluate the association between single biomarkers and breast-feeding duration as a continuous variable (per additional 6 months), we used multivariate linear regression models. Biomarkers were not normally distributed after log-transformation, therefore Box–Cox transformation was used. Associations between breast-feeding and diabetes risk were evaluated using Cox regression modified for the case–cohort design according to the Prentice method [14]. The proportional hazards assumption was tested by plotting the Schönfeld residuals [15]. Age was used as the primary time-dependent variable in all models, with entry time defined as the participant’s age at recruitment, and exit time as the date of diagnosis, death or return of the last follow-up questionnaire. Cox models were stratified for age at baseline and further adjusted for marital status (unmarried, married, divorced, widowed), education (no vocational training or in training, vocational training, technical school, technical college or university), occupation (sedentary, standing, or [heavy] manual work), smoking behaviour (never smoker, ex-smoker, current smoker <20 units/day, current smoker ≥20 units/day), sporting activities (no sport, ≤4 h/week, >4 h/week), biking (no biking, <2.5 h/week, 2.5–4.9 h/week, ≥5 h/week), alcohol intake (no alcohol intake, 0 to ≤5 g/day, >5 to ≤10 g/day, >10 to ≤20 g/day, >20 to ≤40 g/day, >40 g/day), coffee consumption (ml/day), intake of red meat (g/day), intake of whole-grain bread (g/day), age at birth of last child, number of children, duration of oral contraceptive use (no use, ≤5 years, 6–10 years, >10 years), as well as BMI at the age of 25 years, baseline BMI and waist circumference. To evaluate potential biochemical mediators, we adjusted for different biomarkers determined in blood samples collected at baseline. Attenuation of the association indicates a mediator effect. We conducted several sensitivity analyses. We thereby stratified the analysis for number of children, educational level of the mothers, and time since last birth. Stratification for the age at first birth with a cut-off of 25 years was used to evaluate if BMI in young adulthood acts as both a confounder and a mediator. All data analyses were performed using the software package SAS Enterprise Guide 4.3 (SAS Institute, Cary, NC, USA).

### Meta-analysis

We searched the PubMed and Web of Science databases for published studies on the association between breast-feeding and maternal risk of type 2 diabetes. A total of 300 references were identified from the two databases by combining text words and medical subject heading (MESH) terms in PubMed (the search strategy in the ESM Methods and the flow diagram in ESM Fig. 3). Eight additional references were identified by the Web of Science ‘Times cited’ function. The search was completed on 27 March 2014. Reference lists of retrieved studies provided no additional articles. Our inclusion criteria were: prospective cohort study; type 2 diabetes as outcome; description of breast-feeding assessment; presentation of relative risks with 95% CI; description of adjustment for potential confounders. We excluded animal studies and human studies that focused on children’s health or other outcomes such as weight change, metabolic changes, cardiovascular diseases or GDM. Unpublished material was not considered. The literature review was performed by two authors (S. Jäger, S. Jacobs), and data were extracted for multivariate-adjusted models (with and without adjustment for BMI). To evaluate the quality of the included studies, we adapted a score derived from Hu et al [16], which summarizes 14 aspects of each study (ESM Table 1). Meta-analysis was performed with small Stata, version 12.0 (Stata Corp, College Station, TX, USA) using fixed-effects models. Degree of heterogeneity was expressed as an *I* ^2^ statistic, and Cochran’s *Q* test of heterogeneity (α = 0.05) was performed [17]. We assessed potential publication bias by regressing the standard normal deviate (HR/SE) against precision (1/SE) with α = 0.1 [18].

## Results

Table 1 shows baseline characteristics of the sub-cohort by cumulative duration of breast-feeding. Women who breast-fed longer tended to be older and were more likely to be married and better educated. Longer duration of breast-feeding was associated with less smoking and higher physical activity, but occupation and nutritional factors showed no association. Longer duration of breast-feeding was also related to a greater age at birth of the last child, a larger number of children, longer duration of breast-feeding per child, and lower use of oral contraceptive. Women who had never breast-fed had higher BMI and waist circumference levels at the baseline examination compared with women who had. The biomarkers showed no major differences across breast-feeding categories at baseline. Baseline characteristics of the sub-cohort (with and without internal cases) and all incident type 2 diabetes cases are provided in ESM Table 2.

**Table 1:** Baseline characteristics of parous women by duration of breast-feeding in EPIC-Potsdam

Characteristic	Cumulative duration of breast-feeding					*p* value^a^
	0	≤3 weeks	>3 weeks to <2 months	≥2 months to <6 months	≥6 months	
*n*	159	148	184	304	264	
Age at baseline, median (IQR)	47.0 (15.0)	46.0 (15.0)	47.0 (16.5)	47.0 (17.0)	49.0 (19.0)	0.4612
Marital status, %						0.3981
Unmarried	6.92	5.41	4.35	2.30	3.41	
Married	71.1	74.3	77.2	76.6	80.3	
Divorced	17.6	16.2	13.6	16.8	14.4	
Widowed	4.40	4.05	4.89	4.28	1.89	
Education, %						0.0148
No vocational training or in training	4.40	2.03	4.89	3.62	6.82	
Vocational training	41.5	44.6	37.5	35.2	31.8	
Technical school	28.3	33.8	28.3	32.6	25.0	
Technical college, university	25.8	19.6	29.4	28.6	36.4	
Occupation, %						0.1177
Sedentary occupation	65.4	68.9	55.4	63.8	61.7	
Standing occupation and (heavy) manual work	34.6	31.1	44.6	36.2	38.3	
Smoking, %						0.1801
Never smoker	54.7	54.1	57.6	60.5	61.4	
Ex-smoker	25.2	27.0	25.0	20.7	24.2	
Smoker <20 units/day	13.8	14.2	16.9	15.8	11.7	
Smoker ≥20 units/day	6.29	4.73	0.54	2.96	2.65	
Physical activity (h/week), median (IQR)^b^	2.00 (4.00)	1.50 (3.50)	1.50 (3.50)	2.00 (3.50)	2.50 (4.25)	0.0970
Coffee consumption (ml/day), median (IQR)	300 (450)	450 (300)	300 (300)	300 (300)	300 (450)	0.0846
Red meat intake (g/day), median (IQR)	27.4 (27.8)	30.3 (23.2)	33.7 (24.3)	31.3 (26.1)	31.6 (25.2)	0.6453
Whole-grain bread intake (g/day), median (IQR)	30.3 (62.4)	25.7 (61.6)	32.3 (62.1)	29.8 (65.2)	36.9 (62.0)	0.2935
Alcohol (g/day), median (IQR)	5.07 (8.66)	5.36 (10.6)	5.25 (7.82)	5.19 (7.87)	5.02 (8.67)	0.9801
Age at birth of first child, median (IQR)	23.0 (5.00)	23.0 (4.50)	23.0 (4.00)	23.0 (4.00)	23.0 (5.00)	0.0813
Age at birth of last child, mean (SD)	26.6 (5.03)	25.9 (4.12)	26.2 (4.14)	26.9 (4.24)	28.2 (4.14)	<0.0001
Number of children, median (IQR)	1 (1)	2 (1)	2 (1)	2 (1)	2 (1)	<0.0001
Breast-feeding duration per child in months, median (IQR)		0.28 (0.38)	0.81 (0.50)	1.81 (0.97)	4.71 (3.08)	<0.0001
Use of oral contraceptives, %						<0.0001
No use	18.9	12.2	10.9	15.1	17.1	
≤5 years	13.8	14.2	16.9	15.8	28.4	
>5 and ≤10 years	12.0	16.9	18.5	17.1	20.1	
>10 years	55.4	56.8	53.8	52.0	34.5	
BMI at age of 25 years (kg/m^2^), median (IQR)	22.3 (3.28)	21.6 (3.06)	22.1 (2.97)	21.4 (3.04)	21.6 (3.40)	0.0032
BMI at baseline (kg/m^2^), median (IQR)	25.8 (6.56)	24.2 (4.98)	24.9 (5.67)	24.3 (5.25)	24.6 (5.80)	0.0056
Waist circumference (cm), median (IQR)	80.0 (17.0)	78.0 (14.5)	78.3 (16.0)	77.5 (13.8)	79.0 (16.0)	0.0168
Triacylglycerols (mmol/l), median (IQR)	1.20 (0.92)	1.02 (0.59)	1.03 (0.70)	1.09 (0.68)	1.01 (0.62)	0.0060
HDL-cholesterol (mmol/l), mean (SD)	1.52 (0.37)	1.51 (0.39)	1.52 (0.37)	1.57 (0.39)	1.57 (0.37)	0.2561
LDL-cholesterol (mmol/l), mean (SD)	3.00 (0.96)	2.90 (0.79)	2.95 (0.87)	3.06 (0.85)	3.11 (0.95)	0.2215
CRP (nmol/l), median (IQR)	9.90 (25.4)	7.71 (26.0)	9.90 (28.8)	7.71 (18.8)	6.67 (14.4)	0.0697
Fetuin-A (μg/ml), mean (SD)	268 (62.4)	268 (64.9)	274 (60.4)	267 (62.4)	265 (64.6)	0.6200
GGT (μkat/l), median (IQR)	0.23 (0.23)	0.23 (0.20)	0.21 (0.19)	0.21 (0.17)	0.19 (0.20)	0.2433
Adiponectin (μg/ml), median (IQR)	8.52 (5.81)	9.29 (5.20)	9.42 (5.77)	8.93 (5.52)	9.43 (4.78)	0.1943

IQR, interquartile range; GGT, γ-glutamyltransferase

^a^ *χ* ^2^ tests (for categorical variables), ANOVA tests (for normally distributed variables), or Kruskal–Wallis tests (for not normally distributed variables. Variables were log-transformed if this resulted in normal distributions)

^b^Sum of biking and sporting activities in h/week

We used multivariate linear regression models to evaluate the covariate-adjusted associations between different biomarkers and cumulative duration of breast-feeding (ESM Table 3). No single biomarker was strongly associated with breast-feeding duration. However, we observed an inverse relation between breast-feeding duration and triacylglycerols, which became non-significant after adjustment for anthropometry. In contrast, HDL-cholesterol and adiponectin were positively associated.

Table 2 presents associations between duration of breast-feeding and risk of type 2 diabetes. In age-adjusted models, mothers who had ever breast-fed had a lower risk of type 2 diabetes than mothers who had never breast-fed (HR 0.62; 95% CI 0.43, 0.88). This inverse association was also observable when different durations of breast-feeding were evaluated, with women who had breast-fed for ≥6 months having the lowest risk compared with women who had never breast-fed (HR 0.46; 95% CI 0.29, 0.73). Adjustment for lifestyle and other reproductive factors strengthened these associations (HR 0.31 [95% CI 0.18, 0.55] for women who had breast-fed for ≥6 months compared with those who had never breast-fed).

**Table 2:** HRs (95% CI) for type 2 diabetes by duration of breast-feeding, EPIC-Potsdam study

	Ever breast-fed		Cumulative duration of breast-feeding					Per additional 6 months of breast-feeding
	No	Yes	0	≤3 weeks	>3 weeks to <2 months	≥2 months to <6 months	≥6 months	
*n* cases	49	177	49	31	38	66	42	226
Age-adjusted HR (95% CI)	1 (Ref.)	0.62 (0.43, 0.88)	1 (Ref.)	0.76 (0.46, 1.27)	0.65 (0.40, 1.04)	0.67 (0.44, 1.03)	0.46 (0.29, 0.73)	0.84 (0.68, 1.04)
Adjusted HR (95% CI) model I	1	0.66 (0.45, 0.97)	1	0.87 (0.51, 1.51)	0.68 (0.40, 1.15)	0.75 (0.48, 1.20)	0.46 (0.28, 0.76)	0.83 (0.67, 1.03)
Adjusted HR (95% CI) model II	1	0.55 (0.36, 0.82)	1	0.80 (0.46, 1.37)	0.57 (0.33, 0.98)	0.62 (0.39, 1.00)	0.31 (0.18, 0.55)	0.73 (0.56, 0.94)
Adjusted HR (95% CI) model III	1	0.59 (0.39, 0.89)	1	0.87 (0.50, 1.52)	0.57 (0.33, 0.98)	0.69 (0.43, 1.13)	0.34 (0.19, 0.60)	0.73 (0.57, 0.95)
Adjusted HR (95% CI) model IV	1	0.77 (0.47, 1.25)	1	1.16 (0.62, 2.19)	0.77 (0.43, 1.41)	0.82 (0.47, 1.41)	0.47 (0.25, 0.89)	0.80 (0.61, 1.04)

Model I: adjusted for age at baseline, marital status, education, occupation, smoking, sport, cycling, alcohol intake, coffee consumption, intake of red meat, intake of whole-grain bread

Model II: model I + age at birth of last child, number of children, duration of oral contraceptive use

Model III: model II + BMI at age of 25 years

Model IV: model III + BMI at baseline examination, waist circumference at baseline examination

*n* = 1,262

While the lower risk in women who had ever breast-fed compared with women who had never breast-fed remained after BMI at the age of 25 years had been accounted for (HR 0.59; 95% CI 0.39, 0.89), adjustment for BMI and waist circumference at baseline moderately attenuated the association, which became—although still inverse—non-significant (HR 0.77; 95% CI 0.47, 1.25). Similarly, a longer duration of breast-feeding remained associated with a decreased risk of type 2 diabetes after anthropometric characteristics had been accounted for (HR per 6 months 0.80; 95% CI 0.61, 1.04). Women who had breast-fed for 6 months or longer had an HR of 0.47 (95% CI 0.25, 0.89) compared with women who had never breast-fed.

In sensitivity analysis (ESM Table 4), we stratified the data by number of children. Mothers with one child had a confounder-adjusted HR per 6 months of 0.83 (95% CI 0.39, 1.76), with two children they had an HR of 0.77 (95% CI 0.52, 1.15), and with three children the HR was 0.72 (95% CI 0.43, 1.19). We further analysed substrata according to the time lap between last birth and baseline examination (ESM Table 4). The inverse association between breast-feeding and diabetes appeared to be slightly stronger among women who gave birth less than 20 years before baseline (HR per 6 months 0.67; 95% CI 0.31, 1.46) than among women who gave birth to their latest child 20 years or longer before baseline (HR 0.73; 95% CI 0.55, 0.97).

To investigate the role of body mass in early adulthood as a potential confounder vs mediator, we used BMI at age 25 as a proxy measure and stratified the analysis by the age at which women had given birth to their first child (considering an age of either ≤24 or ≥26 to define subgroups) (ESM Table 4). Breast-feeding was associated with lower diabetes risk irrespective of the age at first birth, and adjustment for self-reported BMI at age 25 had no appreciable effect on the strength of association in both strata. In analysis stratified by education, an important confounding factor, we observed no major difference when evaluating breast-feeding categories (ESM Table 4). The HR for duration of breast-feeding per 6 months in women without a university degree was 0.68 (95% CI 0.50, 0.91), while there was no association observable among women with a university degree (HR 0.94; 95% CI 0.59, 1.51).

### Meta-analysis

We conducted a meta-analysis of prospective studies on breast-feeding and diabetes risk. In addition to our study, we were able to identify two previous publications [4, 5], with one article involving two different cohort studies [4] (Table 3). The cohorts included 220,360 mothers from the USA, China and Germany, involving 8,064 incident cases of type 2 diabetes. All studies used self-reported breast-feeding data. However, different breast-feeding categories were defined. We pooled confounder-adjusted data from our cohort and the Nurses’ Health cohorts reported by Stuebe et al [4] in most analyses. The Shanghai Women’s Health cohort [5] was only used for evaluating long duration of breast-feeding (>6–11 months) in comparison with never breast-feeding. However, these data were not adjusted for number of live births. As Fig. 1 indicates, all studies observed inverse associations between breast-feeding and risk of type 2 diabetes. Women who had ever breast-fed were at lower risk than women who had never breast-fed (summary HR 0.95; 95% CI 0.90, 1.00). However, there was significant heterogeneity in the results (*I* ^2^ = 74.3%, *p* = 0.020). The pooled HR was 0.89 (95% CI 0.82, 0.97) for women who had breast-fed for 6–11 months compared with mothers who had never breast-fed (*I* ^2^ = 68.0%, *p* = 0.025). Per additional year of breast-feeding, the pooled HR was 0.93 (95% CI 0.90, 0.96) with high heterogeneity (*I* ^2^ = 88.1%, *p* < 0.001). The Egger test provided evidence of publication bias for two categories of breast-feeding duration (>6–11 months, *p* = 0.022; >11–23 months, *p* = 0.009).

**Table 3:** Prospective studies on breast-feeding and risk of type 2 diabetes

Study	Study population	Study design; follow-up; incident cases	Case ascertainment	Exposure	Exposure categories	Adjustment
Stuebe et al, 2005 [4]	Nurses’ Health Study I, 83,585 mothers	Prospective cohort; follow-up 16 years; 5,145	Self-reported diagnosis	Self-reported total lifetime duration of breast-feeding for all pregnancies in months	No breast-feeding, <1, 1–3, 4–6, 7–11, 12–17, 18–23, 24–35, 36–47, ≥48 months, cannot remember	Age; number of births; nutrition; physical activity; family history of diabetes; smoking status; BMI at age 18 years; birthweight of participants; multivitamin use
	Nurses’ Health Study II, 73,418 mothers	Prospective cohort; follow-up 12 years; 1,132	Self-reported diagnosis	Self-reported total lifetime duration of breast-feeding for all pregnancies in months (1993), self-reported cessation of breast-feeding for each birth (1997 and 2003)	1993: no breast-feeding, <1, 1–3, 4–6, 7–11, 12–17, 18–23, 24–35, 36–47, ≥48 months, cannot remember 1997/2003: 1–2, 3–5, 6–8, 9–11, 12–18, ≥19 months	Age; number of births; nutrition; physical activity; family history of diabetes; smoking status; BMI at age 18 years; birthweight of participants; multivitamin use
Villegas et al, 2008 [5]	Shanghai Women’s Health Study, 62,095 mothers	Prospective cohort; follow-up 4,6 years; 1,561	Self-reported diagnosis	Self-reported breast-feeding duration per child	Calculated categories: no breast-feeding, >0–6, >6–11, >11–35, >36 months	Age; energy intake/day; smoking status; alcohol intake; physical activity; occupation; income; education; hypertension at baseline
Present study, 2014	EPIC-Potsdam Study, 1,262 mothers	Prospective cohort (nested case–cohort) follow-up 7 years; 226	Self-reported diagnosis, confirmed by treating physicians	Self-reported breast-feeding duration per child in months	No breast-feeding, ≤1 week, 2–3 weeks, 4–5 weeks, 6–7 weeks, 2 months, 3 months, 4–5 months, 6–7 months, 8–9 months, 10–11 months, ≥12 months	Age; number of children; nutrition; coffee consumption; physical activity; smoking status; alcohol intake; occupation; education; marital status, oral contraceptive use, age at birth of last child

In a supplemental analysis, we pooled models with adjustment for baseline BMI. While adjustment for BMI had only a marginal effect on the strength of association, associations were mainly not statistically significant (ESM Fig. 4).

### *Analysis of biochemical mediators*

Further adjustment for biomarkers (Table 4) attenuated the association, with the strongest attenuation observed with adjustment for lipid biomarkers (HR per 6 months 0.85; 95% CI 0.66, 1.09) and adiponectin (HR 0.84; 95% CI 0.64, 1.10). Accounting for all biomarkers simultaneously in one model largely attenuated the association (HR per 6 months 0.89; 95% CI 0.68, 1.16; HR for ever vs never breast-fed 0.95; 95% CI 0.59, 1.53).

**Table 4:** HRs (95% CI) for type 2 diabetes by duration of breast-feeding with adjustment for biochemical mediators, EPIC-Potsdam study

	Ever breast-fed		Cumulative duration of breast-feeding					
	No	Yes	0	≤3 weeks	>3 weeks to <2 months	≥2 months to <6 months	≥6 months	Per additional 6 months of breast-feeding
*n* cases	49	177	49	31	38	66	42	226
Model I	1	0.77 (0.47, 1.25)	1	1.16 (0.62, 2.19)	0.77 (0.43, 1.41)	0.82 (0.47, 1.41)	0.47 (0.25, 0.89)	0.80 (0.61, 1.04)
Model I + HDL, LDL, triacylglycerols	1	0.88 (0.55, 1.42)	1	1.38 (0.71, 2.69)	0.93 (0.51, 1.67)	0.91 (0.53, 1.57)	0.55 (0.29, 1.02)	0.85 (0.66, 1.09)
Model I + CRP	1	0.73 (0.45, 1.18)	1	1.11 (0.59, 2.08)	0.72 (0.39, 1.32)	0.77 (0.45, 1.33)	0.47 (0.25, 0.88)	0.81 (0.62, 1.06)
Model I + fetuin-A, GGT	1	0.78 (0.48, 1.26)	1	1.24 (0.66, 2.33)	0.78 (0.43, 1.42)	0.83 (0.48, 1.42)	0.47 (0.24, 0.90)	0.81 (0.62, 1.06)
Model I + adiponectin	1	0.91 (0.57, 1.45)	1	1.64 (0.90, 3.00)	0.91 (0.51, 1.64)	0.93 (0.54, 1.59)	0.58 (0.31, 1.07)	0.84 (0.64, 1.10)
Model I + HDL, LDL, triacylglycerols + CRP + fetuin-A, GGT + adiponectin	1	0.95 (0.59, 1.53)	1	1.74 (0.91, 3.32)	1.00 (0.55, 1.83)	0.91 (0.53, 1.58)	0.62 (0.33, 1.16)	0.89 (0.68, 1.16)

GGT, γ-glutamyltransferase

Model I adjusted for age at baseline, marital status, education, occupation, smoking, sport, cycling, alcohol intake, coffee consumption, intake of red meat, intake of whole-grain bread, age at birth of last child, number of children, duration of oral contraceptive use, BMI at age of 25 years, BMI and waist circumference at baseline examination. *n* = 1,262

## Discussion

In the present study, breast-feeding was associated with a lower risk of type 2 diabetes. This association was independent of potential confounding sociodemographic, lifestyle and reproductive risk factors. Furthermore, meta-analysis of cohort studies indicated an inverse association. Adjustment for BMI at baseline had little effect on this relationship, while accounting for an at-risk metabolic profile in adult life, reflected by several biomarkers, largely explained the association between breast-feeding and type 2 diabetes.

A systematic literature search and meta-analysis including results from EPIC-Potsdam as well as findings from previous prospective cohort studies [4, 5] suggest that longer breast-feeding duration may be associated with a lower risk of type 2 diabetes. Included studies showed good quality characterised by their prospective design, a comparable breast-feeding assessment, detailed adjustment sets and low rates of loss to follow-up (ESM Table 1). However, there was high heterogeneity between the studies, which complicates the drawing of general conclusions. The women in these four cohorts showed quite different breast-feeding patterns. German women breast-fed for a comparatively short time. Further limitations come from the imprecision of assessing breast-feeding behaviour on the basis of self-reports. Some studies found good validity of self-reported breast-feeding history after more than 20 years compared with medical records [19, 20]. Others criticise the retrospective collection of breast-feeding data via questionnaires [21]. Factors such as socioeconomic status and the number of children could affect the mother's memory [22–24]. Furthermore, breast-feeding was self-reported, irrespective of additional feeding, and therefore was not stratified as exclusive or non-exclusive breast-feeding, which may be of importance when evaluating short breast-feeding periods [25].

We cannot exclude residual confounding. Breast-feeding is highly related to sociodemographic factors. Adjusting for marital status, educational level and occupation and stratification for educational level had no influence on the association in our study. However, we cannot rule out the possibility that other correlates of breast-feeding, e.g. related to income, could explain our observation.

In addition, there is the possibility that cases remained undiagnosed during follow-up and were therefore misclassified as false-negative. However, this misclassification should not bias the associations given that false-positive case definitions should have been rare in most cohorts because of the verification procedures (EPIC-Potsdam) or the high accuracy of self-reports among nurses (US cohorts) [26].

Finally, the Egger test provided evidence of publication bias. We addressed this observation by using fixed-effects models in the meta-analysis [27]. However, only a few studies were available for this meta-analysis, and exclusion of one study that contributed the lowest weight but showed the strongest associations did not alter the overall result of the meta-analysis (data not shown).

The underlying mechanisms by which breast-feeding could lower diabetes risk are still unknown, although several hypotheses of dependence on weight development and metabolic pathways have been suggested.

High prepregnancy BMI and extreme weight gain during pregnancy have been associated with early termination of breast-feeding [28, 29]. Therefore, a shorter duration of breast-feeding may be a consequence, rather than a cause, of increased body weight, supporting the hypothesis that BMI acts as a confounder. However, other studies suggest that weight changes may mediate the association between breast-feeding and incident type 2 diabetes [6]. Breast-feeding is associated with higher energy requirements [7]. These requirements are compensated for by a higher energy intake and less physical activity during the first 3 months after delivery. Later on, women begin to mobilise fat stores accumulated through pregnancy [30, 31]. During breast-feeding periods, significantly higher lipolysis in the femoral region has been observed [32]. Therefore femoral adipose tissue acts as an important source of energy during lactation. Previous studies have obtained controversial results on the importance of weight change. Butte and Hopkinson [8] reviewed 17 prospective studies conducted in industrialised and developing countries. Most studies demonstrated no difference in weight change between breast-feeding and non-breast-feeding women. However, participants were only followed-up for up to 6 months post partum. While some studies support these findings [33], others found breast-feeding to be associated with lower post partum weight retention [10, 34]. In our study, adjusting for self-reported BMI at the age of 25 showed only a slight effect on the relationship. Although adjustment for BMI and waist circumference assessed at baseline had stronger attenuating effects, an inverse association between breast-feeding and diabetes remained, especially in the longest-duration breast-feeding category. It can be discussed whether BMI assessed many years after breast-feeding acts as a confounder or a mediator in this context. Although less weight gain with breast-feeding is a plausible mediator, BMI at baseline may also partly reflect prepregnancy weight status or other confounding factors associated with body fatness and which may have insufficiently been controlled for by using lifestyle confounders and retrospectively self-reported BMI at age 25.

While adjustment for biomarkers revealed an attenuation of results in our analysis, another cohort study found no clear dose–response associations between breast-feeding and maternal glucose and lipid metabolism or inflammatory markers 3 years post partum [35].

Still, prolonged breast-feeding seems to affect lipoprotein profiles. In a 3-year prospective study, breast-feeding women had higher HDL-cholesterol levels than non-breast-feeding mothers [9]. Others reported a decline in triacylglycerols after delivery, which was more rapid in breast-feeding mothers [36]. However, at the end of the breast-feeding period, blood lipids had again reached their baseline level [37]. We found a weak association between breast-feeding duration and triacylglycerols as well as with HDL-cholesterol in adult life in covariate-adjusted linear regression models. Accounting for them in Cox models attenuated the association of breast-feeding with diabetes.

In addition, we investigated mediating effects related to insulin sensitivity during adulthood. Thereby we saw a positive association between cumulative breast-feeding duration and adiponectin levels. Although we observed an attenuation by accounting for adiponectin in our mediator analysis, others did not find a linear association between breast-feeding duration and adiponectin levels 3 years post partum [38]. One study found lower fasting insulin levels in breast-feeding than non-breast-feeding women at 6 months post partum, although there was no difference in fasting glucose levels [7]. Others observed a trend for increased fasting insulin levels and HOMA-IR in non-breast-feeding compared with breast-feeding mothers [9]. Animal studies support this. During lactation, the insulin sensitivity changes are tissue- specific [39] as a result of alterations in signal transmission after binding of insulin to its receptor [40]. For instance, the number of insulin receptors of mammary epithelial cells is increased in mice [41]. Therefore the mammary gland is more insulin-sensitive than adipose tissue or muscle to ensure an adequate supply of nutrients during lactation [39, 42].

The long time lag (>20 years) between the last breast-feeding period and blood collection at the baseline examination might mask favourable effects of breast-feeding on metabolic variables in our analysis. However, adjustment for biomarkers still largely attenuated the association between breast-feeding and diabetes in our study. This suggests that breast-feeding leads to an overall more favourable metabolic risk profile in the long term. Previous studies discussed an effect of breast-feeding on long-term weight development as the main factor for decreased diabetes risk. However, our study revealed that BMI at baseline examination only partly explains this association.

In conclusion, the evidence from this study and previous studies, summarised by means of meta-analysis, suggests that longer breast-feeding duration may be related to lower maternal type 2 diabetes risk. However, the role of body weight as a mediator or confounder remains uncertain.

## Electronic supplementary material

Below is the link to the electronic supplementary material.

ESM Methods (PDF 5.61 kb)

ESM Fig. 1 (PDF 51 kb)

ESM Fig. 2 (PDF 201 kb)

ESM Fig. 3 (PDF 108 kb)

ESM Fig. 4 (PDF 102 kb)

ESM Table 1 (PDF 95 kb)

ESM Table 2 (PDF 91 kb)

ESM Table 3 (PDF 82 kb)

ESM Table 4 (PDF 151 kb)

## Abbreviations

CRP: C-reactive protein
EPIC: European Prospective Investigation into Cancer and Nutrition
GDM: Gestational diabetes mellitus
