# Supplementary material for: Breast-feeding and maternal risk of type 2 diabetes: a prospective study and meta-analysis
Source: Diabetologia. 2014 May 1;57(7):1355–65. doi: 10.1007/s00125-014-3247-3 (PMC4052010; doi:10.1007/s00125-014-3247-3)
Supplement: Supplementary file 1 — (PDF 5.61 kb) [file 125_2014_3247_MOESM1_ESM.pdf]

PUBMED Search Strategy (search on 27 March 2014)

Search: (((((((glucose) OR (insulin) OR (glycosylated haemoglobin) OR (glycosylated hemoglobin) OR (glycated haemoglobin) OR (glycated hemoglobin) OR (homeostasis model assessment) OR (homa) OR (HbA1) OR (HbA1c) OR (diabetes type 2))) OR ("Diabetes Mellitus, Type 2/epidemiology"[Mesh] OR "Blood Glucose"[Mesh] OR "Glucose Metabolism Disorders"[Mesh]))) AND ((((("Breast Feeding"[Mesh] OR "Lactation/physiology"[Mesh])) OR ((breastfeed*) OR (lactation)))) AND ("Humans"[Mesh] AND "Adult"[Mesh] AND "Female"[Mesh]))) AND ("Cohort Studies"[Mesh] OR "incidence"[Mesh])) NOT (type 1 diabetes mellitus)

Web of Science Search Strategy (search on 27 March 2014)

TS= (((breast-feed* OR breastfeed* OR lactation)) AND ((glucose) OR (insulin) OR ("glycosylated haemoglobin") OR ("glycosylated hemoglobin") OR ("glycated haemoglobin") OR ("glycated hemoglobin") OR ("homeostasis model assessment") OR (homa) OR (HbA1) OR (HbA1c) OR (diabetes type 2)) AND (("Cohort Study") OR (incidence) OR (prospective)) NOT ("type 1"))
