# Supplementary material for: Breast-feeding and maternal risk of type 2 diabetes: a prospective study and meta-analysis
Source: Diabetologia. 2014 May 1;57(7):1355–65. doi: 10.1007/s00125-014-3247-3 (PMC4052010; doi:10.1007/s00125-014-3247-3)
Supplement: Supplementary file 2 — (PDF 51 kb) [file 125_2014_3247_MOESM2_ESM.pdf]

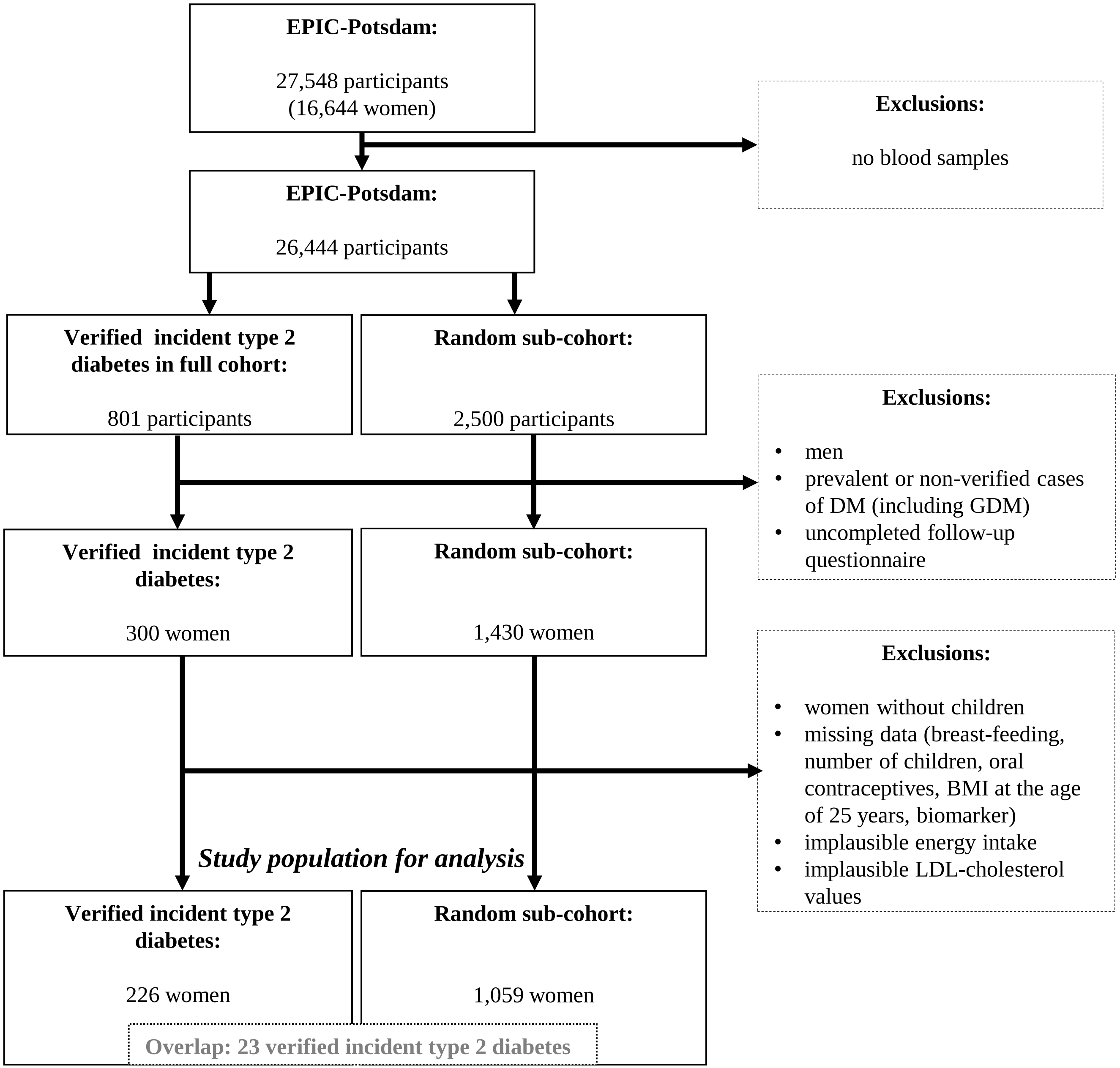


**ESM Figure 1** Case-cohort design within the EPIC-Potsdam study

GDM, gestational diabetes mellitus; DM, diabetes mellitus
