# Supplementary material for: Breast-feeding and maternal risk of type 2 diabetes: a prospective study and meta-analysis
Source: Diabetologia. 2014 May 1;57(7):1355–65. doi: 10.1007/s00125-014-3247-3 (PMC4052010; doi:10.1007/s00125-014-3247-3)
Supplement: Supplementary file 3 — (PDF 201 kb) [file 125_2014_3247_MOESM3_ESM.pdf]

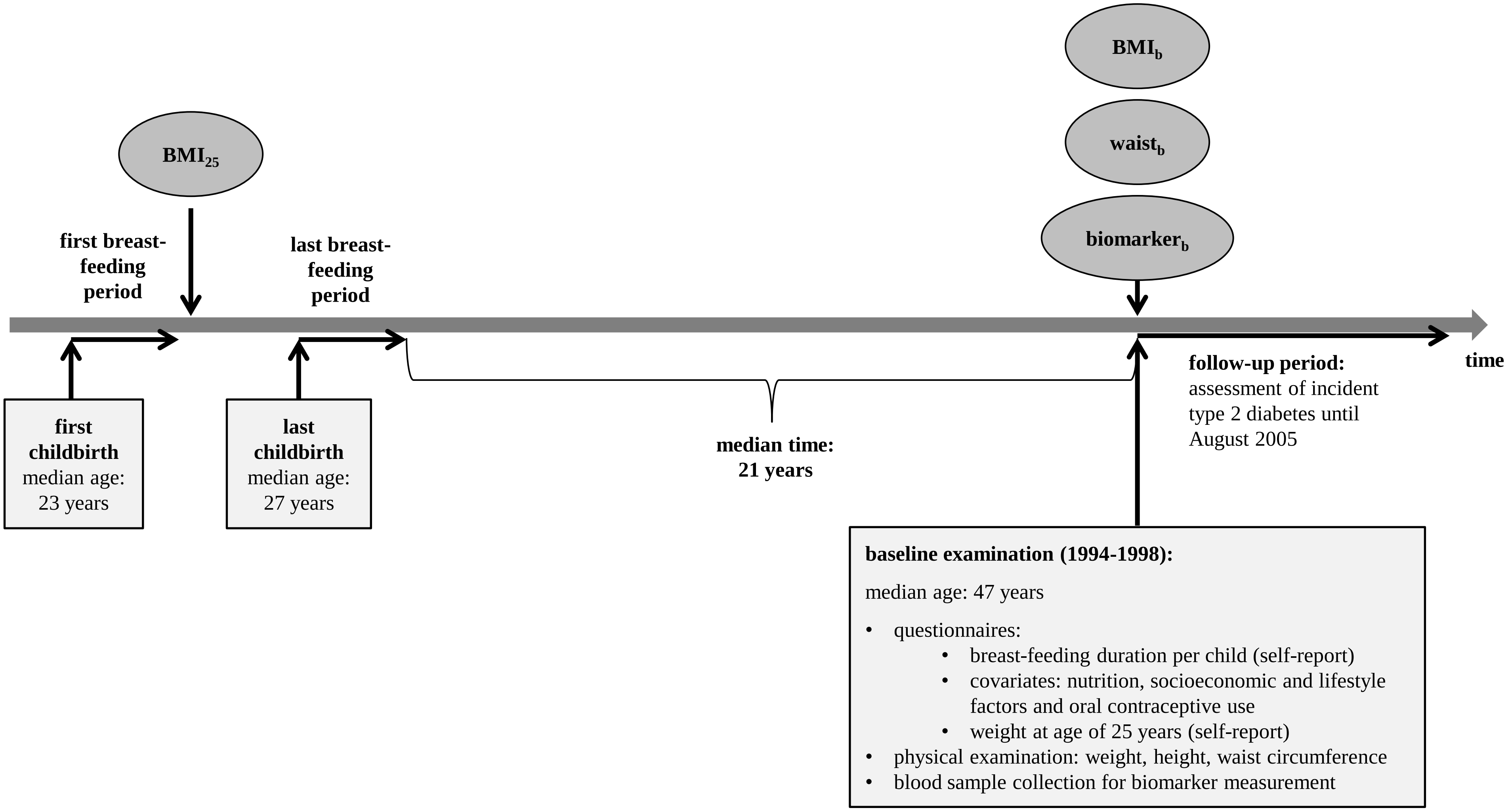

ESM Figure 2 Study design and the time points of data assessment

BMI$_{25}$, BMI at age of 25 years; BMI$_b$, BMI at baseline; waist$_b$, waist circumference at baseline; biomarker$_b$, measured biomarker levels in blood samples collected at baseline
