# Supplementary figures and images for: Breast-feeding and maternal risk of type 2 diabetes: a prospective study and meta-analysis
Source: Diabetologia. 2014 May 1;57(7):1355–65. doi: 10.1007/s00125-014-3247-3 (PMC4052010; doi:10.1007/s00125-014-3247-3)

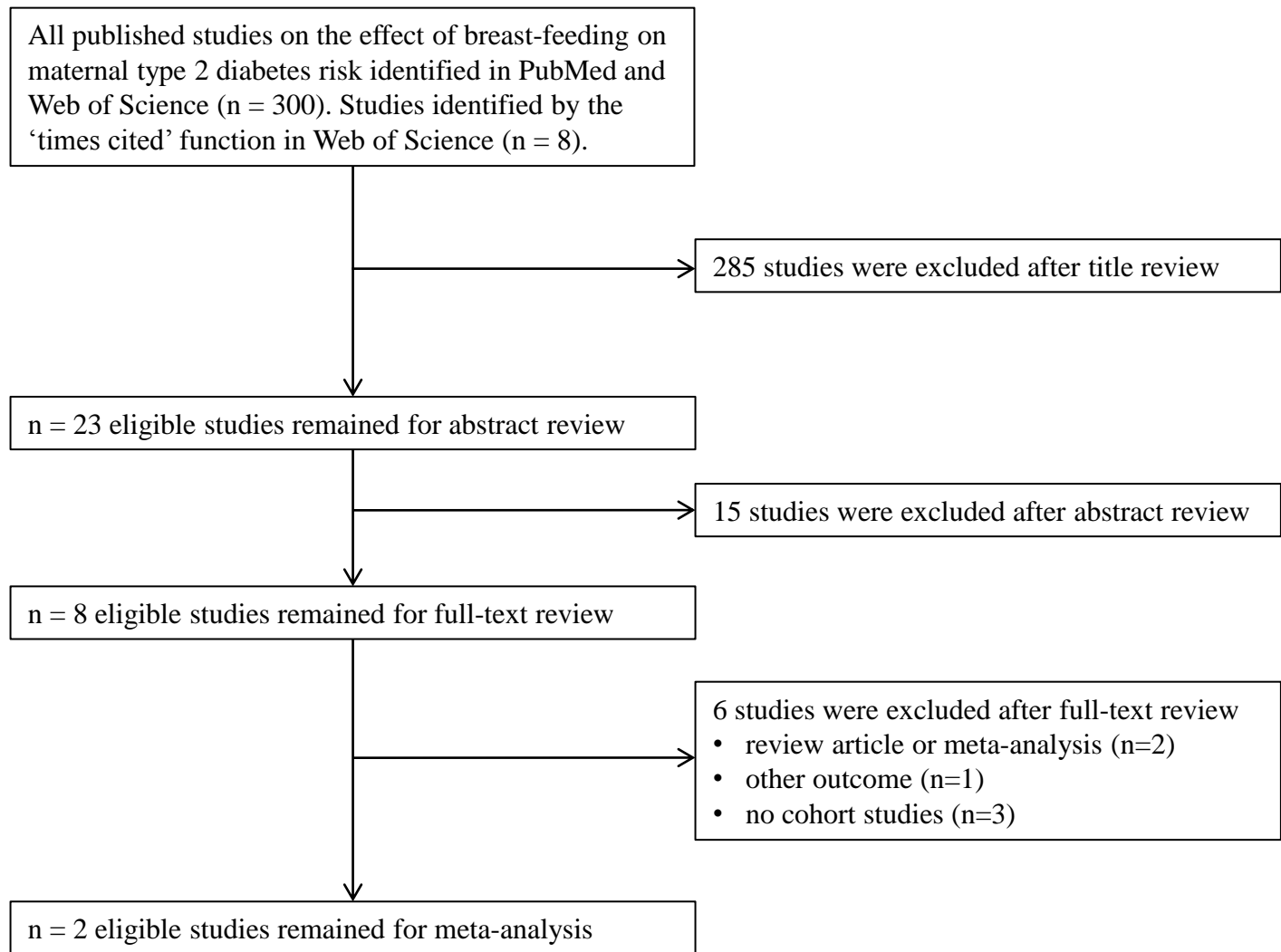

**ESM Figure 3** Flow diagram of selection of studies for the meta-analysis

Supplement: Supplementary file 4 — (PDF 108 kb) [file 125_2014_3247_MOESM4_ESM.pdf]
