# Supplementary material for: Breast-feeding and maternal risk of type 2 diabetes: a prospective study and meta-analysis
Source: Diabetologia. 2014 May 1;57(7):1355–65. doi: 10.1007/s00125-014-3247-3 (PMC4052010; doi:10.1007/s00125-014-3247-3)
Supplement: Supplementary file 5 — (PDF 102 kb) [file 125_2014_3247_MOESM5_ESM.pdf]

ESM Figure 4 HRs (95% CIs) for association between duration of breast-feeding and type 2 diabetes.

Categories of breast-feeding duration (compared with no breast-feeding): breast-feeding vs non-breast-feeding (**a**), >0 to 3 months (**b**), >3 to 6 months (**c**), >6 to 11 months (**d**), >11 to 23 months (**e**), per additional year (**f**); Models are adjusted for potential confounders and baseline BMI. NHS, Nurses' Health Study; SWHS, Shanghai Women's Health Study

a

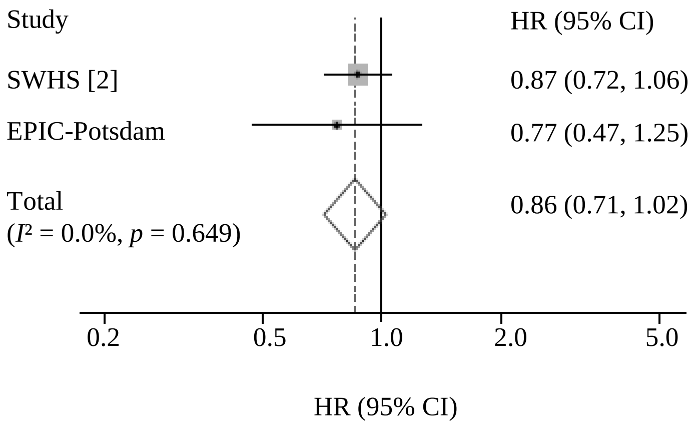
Study
HR (95% CI)
SWHS [2]
0.87 (0.72, 1.06)
EPIC-Potsdam
0.77 (0.47, 1.25)
Total
(I² = 0.0%, p = 0.649)
0.86 (0.71, 1.02)
0.2
0.5
1.0
2.0
5.0
HR (95% CI)

b

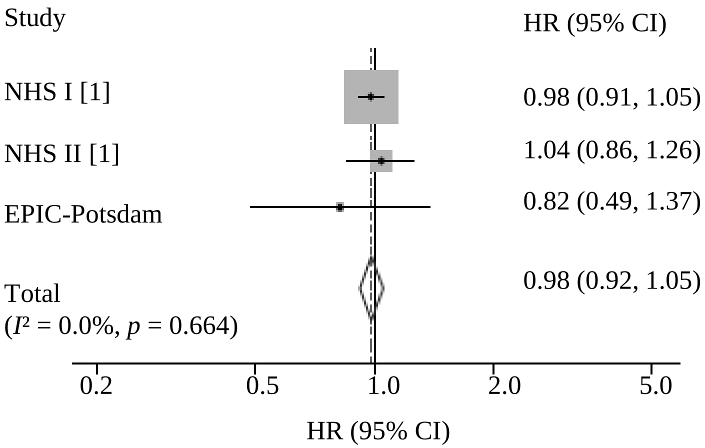
Study
HR (95% CI)
NHS I [1]
0.98 (0.91, 1.05)
NHS II [1]
1.04 (0.86, 1.26)
EPIC-Potsdam
0.82 (0.49, 1.37)
Total
(I^2 = 0.0%, p = 0.664)
0.98 (0.92, 1.05)
0.2
0.5
1.0
2.0
5.0
HR (95% CI)

c

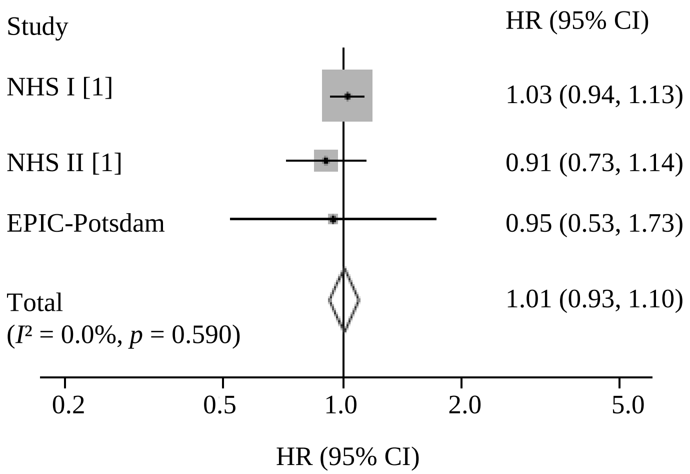
Study
HR (95% CI)
NHS I [1]
1.03 (0.94, 1.13)
NHS II [1]
0.91 (0.73, 1.14)
EPIC-Potsdam
0.95 (0.53, 1.73)
Total
(I² = 0.0%, p = 0.590)
1.01 (0.93, 1.10)
0.2
0.5
1.0
2.0
5.0
HR (95% CI)

d

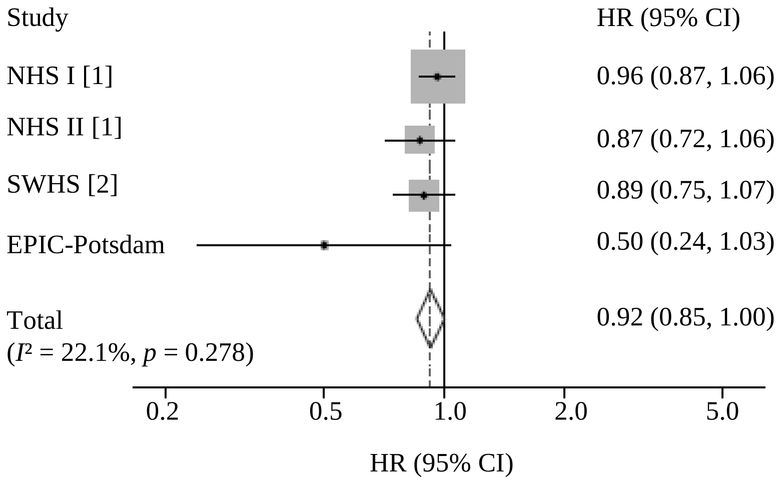
Study
HR (95% CI)
NHS I [1]
0.96 (0.87, 1.06)
NHS II [1]
0.87 (0.72, 1.06)
SWHS [2]
0.89 (0.75, 1.07)
EPIC-Potsdam
0.50 (0.24, 1.03)
Total
(I^2 = 22.1%, p = 0.278)
0.92 (0.85, 1.00)
0.2
0.5
1.0
2.0
5.0
HR (95% CI)

e

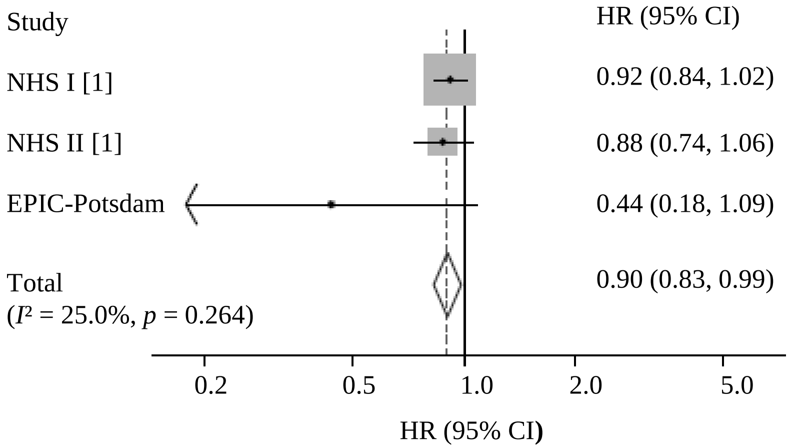
Study
HR (95% CI)
NHS I [1]
0.92 (0.84, 1.02)
NHS II [1]
0.88 (0.74, 1.06)
EPIC-Potsdam
0.44 (0.18, 1.09)
Total
(I^2 = 25.0%, p = 0.264)
0.90 (0.83, 0.99)
0.2
0.5
1.0
2.0
5.0
HR (95% CI)

f

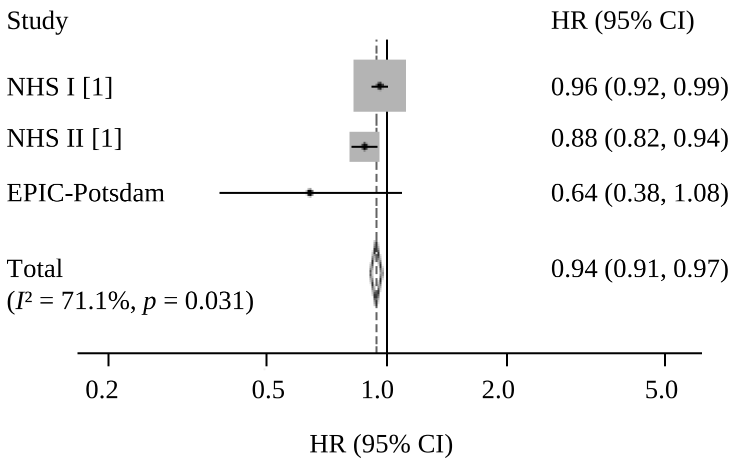
Study
HR (95% CI)
NHS I [1]
0.96 (0.92, 0.99)
NHS II [1]
0.88 (0.82, 0.94)
EPIC-Potsdam
0.64 (0.38, 1.08)
Total
(I^2 = 71.1%, p = 0.031)
0.94 (0.91, 0.97)
0.2
0.5
1.0
2.0
5.0
HR (95% CI)

References

[1] Stuebe AM, Rich-Edwards JW, Willett WC, Manson JE, Michels KB (2005) Duration of lactation and incidence of type 2 diabetes. JAMA 294: 2601-2610

[2] Villegas R, Gao YT, Yang G, et al. (2008) Duration of breast-feeding and the incidence of type 2 diabetes mellitus in the Shanghai Women's Health Study. Diabetologia 51: 258-266
