# Supplementary material for: Breast-feeding and maternal risk of type 2 diabetes: a prospective study and meta-analysis
Source: Diabetologia. 2014 May 1;57(7):1355–65. doi: 10.1007/s00125-014-3247-3 (PMC4052010; doi:10.1007/s00125-014-3247-3)
Supplement: Supplementary file 6 — (PDF 95 kb) [file 125_2014_3247_MOESM6_ESM.pdf]

ESM Table 1 Quality assessment of prospective cohort studies on breast-feeding and diabetes risk

		1	2	3	4	5	6	7	8
Study		Are the comparison groups from the same source population?	Is loss to follow-up rate < 20%?	Is breast-feeding duration assessed by questionnaires?	Is duration of exclusive breast-feeding duration available?	Is duration of breast-feeding assessed per child?	Is there a validation study for assessing validity of self-reported breast-feeding duration?	Are breast-feeding data assessments updated during follow-up?	Are self-reported diabetes cases confirmed by medical records, physicians or validated questionnaires?
Stuebe et al, 2005 [1]	NHS I	1	1	1	0	0	0	0	1
	NHS II	1	1	1	0	1	0	1	1
Villegas et al, 2008 [2]	SWHS	1	1	1	0	1	0	0	0
present study, 2014	EPIC-Potsdam	1	1	1	0	1	0	0	1

Study		9	10	11	12	13	14	
Study		Are prevalent diabetes cases excluded at baseline?	Is an accurate date of diagnosis available for diabetes cases?	Is the total follow-up duration ≥ 5 years	Is Cox regression used to model associations between breast-feeding duration and diabetes risk?	Are age, nutritional factors and lifestyle practices controlled for in the statistical analysis?	Are reproductive factors controlled for in the statistical analysis? (e.g. number of children)	Overall quality score
Stuebe et al, 2005 [1]	NHS I	1	1	1	1	1	1	10
	NHS II	1	1	1	1	1	1	12
Villegas et al, 2008 [2]	SWHS	1	1	0	1	1	0	8
present study, 2014	EPIC-Potsdam	1	1	1	1	1	1	11

NHS, Nurses' Health Study; SWHS, Shanghai Women's Health Study
This score was adapted from Hu et al. [3]

References

[1] Stuebe AM, Rich-Edwards JW, Willett WC, Manson JE, Michels KB (2005) Duration of lactation and incidence of type 2 diabetes. JAMA 294: 2601-2610

[2] Villegas R, Gao YT, Yang G, et al. (2008) Duration of breast-feeding and the incidence of type 2 diabetes mellitus in the Shanghai Women's Health Study. Diabetologia 51: 258-266

[3] Hu EA, Pan A, Malik V, Sun Q (2012) White rice consumption and risk of type 2 diabetes: meta-analysis and systematic review. BMJ 344: e1454-e1462
