# Supplementary material for: Breast-feeding and maternal risk of type 2 diabetes: a prospective study and meta-analysis
Source: Diabetologia. 2014 May 1;57(7):1355–65. doi: 10.1007/s00125-014-3247-3 (PMC4052010; doi:10.1007/s00125-014-3247-3)
Supplement: Supplementary file 7 — (PDF 91 kb) [file 125_2014_3247_MOESM7_ESM.pdf]

ESM Table 2 Baseline characteristics of the EPIC-Potsdam case-cohort

Characteristics	Sub-cohort (inclusive internal cases)	Sub-cohort (exclusive internal cases)	All incident type 2 diabetes cases
n	1059	1036	226
Age at baseline, median (IQR)	47.0 (17.0)	47.0 (16.0)	56.0 (11.0)
Marital status, %			
unmarried	4.06	4.15	2.65
married	76.5	76.6	73.9
divorced	15.7	15.5	17.7
widowed	3.78	3.67	5.75
Education, %			
no vocational training or in training	4.53	4.44	10.62
vocational training	37.0	36.5	46.5
technical school	29.5	29.6	25.7
technical college, university	29.0	29.4	17.3
Occupation, %			
sedentary occupation	62.8	62.6	61.1
standing occupation and (heavy) manual work	37.2	37.4	38.9
Smoking, %			
never smoker	58.5	58.9	56.6
ex-smoker	23.9	23.7	30.1
smoker < 20 units/day	14.5	14.4	8.85
smoker ≥ 20 units/day	3.21	3.09	4.42
Physical Activity (h/week), median (IQR)[a]	2.00 (4.00)	2.00 (4.00)	1.50 (3.50)
Coffee consumption (ml/day), median (IQR)	300 (386)	300 (300)	300 (300)
Red meat intake (g/day), median (IQR)	31.3 (25.5)	31.3 (25.5)	34.8 (24.6)
Whole-grain bread intake (g/day), median (IQR)	32.6 (63.2)	33.2 (63.5)	30.9 (60.9)
Alcohol (g/day), median (IQR)	5.16 (8.41)	5.16 (8.44)	3.78 (7.55)
Age at birth of first child, median (IQR)	23.0 (4.00)	23.0 (4.00)	22.0 (5.00)
Age at birth of last child, mean (SD)	26.9 (4.38)	26.9 (4.39)	27.1 (4.57)
Number of children, median (IQR)	2 (1)	2 (1)	2 (2)
Use of oral contraceptives, %			
no use	15.0	14.7	23.0
≤ 5 years	18.6	18.6	20.8
> 5 and ≤ 10 years	17.3	17.3	17.3
> 10 years	49.1	49.4	38.9
BMI at age of 25 years (kg/m^2), median (IQR)	21.7 (3.18)	21.6 (3.09)	22.7 (4.13)
BMI at baseline (kg/m^2), median (IQR)	24.6 (5.56)	24.6 (5.41)	30.4 (6.35)
Waist circumference (cm), median (IQR)	78.0 (15.0)	78.0 (14.5)	94.0 (15.5)

IQR, interquartile range

[a] Sum of biking and sporting activities in h/week.

A random sub-cohort of 2,500 individuals was selected from all participants with collected blood of the EPIC-Potsdam study population. Additionally, 849 incident type 2 diabetes cases were identified in the full cohort. 801 cases provided blood samples. After application of similar exclusion criteria 1059 women in the sub-cohort and 226 incident cases remained for this analysis. Because the sub-cohort is representative of the full cohort at baseline in case-cohort studies, the sub-cohort in our analyses included 23 women who developed incident type 2 diabetes during follow-up. These cases are termed as internal cases.
