# Supplementary material for: Breast-feeding and maternal risk of type 2 diabetes: a prospective study and meta-analysis
Source: Diabetologia. 2014 May 1;57(7):1355–65. doi: 10.1007/s00125-014-3247-3 (PMC4052010; doi:10.1007/s00125-014-3247-3)
Supplement: Supplementary file 8 — (PDF 82 kb) [file 125_2014_3247_MOESM8_ESM.pdf]

ESM Table 3 Association of breast-feeding with biomarker levels at baseline

**Biomarker**	**Age-adjusted model**		**Covariate-adjusted model I**		**Covariate-adjusted model II**	
	β (95% CI)	Adj. R^2	β (95% CI)	Adj. R^2	β (95% CI)	Adj. R^2
Triacylglycerols (mmol/l)	-0.084 (-0.142, -0.026)	0.0690	-0.077 (-0.142, -0.013)	0.1041	-0.057 (-0.117, 0.003)	0.2235
HDL-cholesterol (mmol/l)	0.044 (-0.017, 0.104)	0.0001	0.061 (-0.006, 0.128)	0.0409	0.044 (-0.020, 0.108)	0.1145
LDL-cholesterol (mmol/l)	0.038 (-0.017, 0.094)	0.1550	0.027 (-0.035, 0.089)	0.1735	0.032 (-0.030, 0.094)	0.1791
CRP (nmol/l)	-0.082 (-0.141, -0.022)	0.0138	-0.052 (-0.117, 0.012)	0.0996	-0.027 (-0.085, 0.032)	0.2630
Fetuin-A (µg/ml)	-0.018 (-0.077, 0.042)	0.0288	-0.023 (-0.089, 0.044)	0.0392	-0.016 (-0.083, 0.050)	0.0480
GGT (µkat/l)	-0.032 (-0.092, 0.027)	0.0286	-0.046 (-0.112, 0.020)	0.0589	-0.029 (-0.092, 0.034)	0.1442
Adiponectin (µg/ml)	0.029 (-0.031, 0.088)	0.0217	0.054 (-0.013, 0.120)	0.0511	0.039 (-0.024, 0.103)	0.1359

GGT, γ-glutamyltransferase.

Covariate model I was adjusted for age at baseline, marital status, education, occupation, smoking, sport, cycling, alcohol intake, coffee consumption, intake of red meat, intake of whole-grain bread, age at birth of last child, number of children, duration of oral contraceptive use.

Covariate model II was further adjusted for BMI at baseline and waist circumference at baseline. Biomarkers were not normally distributed and were transformed using Box-Cox transformation before analysis; Associations were quantified with standardized beta coefficients.

n= 1059.
