# Supplementary material for: Breast-feeding and maternal risk of type 2 diabetes: a prospective study and meta-analysis
Source: Diabetologia. 2014 May 1;57(7):1355–65. doi: 10.1007/s00125-014-3247-3 (PMC4052010; doi:10.1007/s00125-014-3247-3)
Supplement: Supplementary file 9 — (PDF 151 kb) [file 125_2014_3247_MOESM9_ESM.pdf]

ESM Table 4 HRs (95% CI) for type 2 diabetes by duration of breast-feeding stratified for number of children, time since last birth, age at birth of first child and educational level

		**Cumulative duration of breast-feeding**					
		**0**	**≤ 3 weeks**	**> 3 weeks to < 2 months**	**≥ 2 months to < 6 months**	**≥ 6 months**	**per additional 6 months of breast-feeding**
One child	n (cases)	19	9	14	18	5	65
	Covariate-adjusted HR[a]	1 (Ref.)	0.77 (0.29, 2.04)	0.69 (0.24, 2.00)	1.04 (0.46, 2.37)	0.49 (0.16, 1.48)	0.83 (0.39, 1.76)
Two children	n (cases)	20	16	14	33	20	103
	Covariate - adjusted HR[a]	1 (Ref.)	0.66 (0.29, 1.47)	0.42 (0.18, 0.95)	0.62 (0.30, 1.26)	0.31 (0.13, 0.73)	0.77 (0.52, 1.15)
Three or more children	n (cases)	10	6	10	15	17	58
	Covariate - adjusted HR[a]	1 (Ref.)	2.15 (0.16, 29.1)	0.18 (0.02, 1.43)	0.12 (0.02, 0.72)	0.07 (0.01, 0.47)	0.72 (0.43, 1.19)
Time since last birth < 20 years	n (cases)	13	11	7	11	6	48
	Covariate - adjusted HR[b]	1 (Ref.)	0.68 (0.27, 1.75)	0.23 (0.06, 0.82)	0.27 (0.10, 0.73)	0.27 (0.09, 0.77)	0.67 (0.31, 1.46)
Time since last birth ≥ 20 years	n (cases)	36	20	31	55	36	178
	Covariate - adjusted HR[b]	1 (Ref.)	0.76 (0.38, 1.52)	0.67 (0.36, 1.26)	0.76 (0.43, 1.33)	0.32 (0.16, 0.63)	0.73 (0.55, 0.97)
Age at birth of first child ≤ 24 years[c]	n (cases)	27	25	29	52	34	167
	Covariate-adjusted HR[b]	1 (Ref.)	0.96 (0.50, 1.83)	0.64 (0.34, 1.19)	0.72 (0.41, 1.27)	0.33 (0.17, 0.65)	0.74 (0.55, 1.00)
	+ BMI at age 25	1 (Ref.)	1.08 (0.56, 2.09)	0.67 (0.36, 1.24)	0.82 (0.46, 1.46)	0.35 (0.18, 0.69)	0.72 (0.53, 0.99)
Age at birth of first child ≥ 26 years[c]	n (cases)	20	3	7	10	4	44
	Covariate - adjusted HR[b]	1 (Ref.)	0.19 (0.04, 0.97)	0.29 (0.07, 1.17)	0.24 (0.06, 1.06)	0.09 (0.02, 0.36)	0.40 (0.19, 0.85)
	+ BMI at age 25	1 (Ref.)	0.25 (0.04, 1.39)	0.24 (0.05, 1.13)	0.43 (0.09, 1.95)	0.10 (0.02, 0.45)	0.47 (0.22, 1.02)

		Cumulative duration of breast-feeding				
		**0**	**< 2 months**	**≥ 2 months to < 6 months**	**≥ 6 months**	**per additional 6 months of breast-feeding**
University degree	n (cases)	9	9	10	11	39
	Covariate-adjusted HR[d]	1 (Ref.)	0.45 (0.12, 1.69)	0.37 (0.07, 2.04)	0.44 (0.13, 1.50)	0.94 (0.59, 1.51)
No university degree	n (cases)	40	60	56	31	187
	Covariate - adjusted HR[d]	1 (Ref.)	0.75 (0.45, 1.25)	0.72 (0.42, 1.24)	0.28 (0.15, 0.54)	0.68 (0.50, 0.91)

[a] Adjusted for age at baseline, marital status, education, occupation, smoking, sport, cycling, alcohol intake, coffee consumption, intake of red meat, intake of whole-grain bread, age at birth of last child, duration of oral contraceptive use.

[b] Adjusted for age at baseline, marital status, education, occupation, smoking, sport, cycling, alcohol intake, coffee consumption, intake of red meat, intake of whole-grain bread, number of children, duration of oral contraceptive use.

[c] case-cohort with 1161 women (211 cases); 101 Women who gave first birth at the age of 25 were excluded from original case-cohort.

[d] Adjusted for age at baseline, marital status, occupation, smoking, sport, cycling, alcohol intake, coffee consumption, intake of red meat, intake of whole-grain bread, age at birth of last child, number of children, duration of oral contraceptive use.
